# Burn Injury Alters the Intestinal Microbiome and Increases Gut Permeability and Bacterial Translocation

**DOI:** 10.1371/journal.pone.0129996

**Published:** 2015-07-08

**Authors:** Zachary M. Earley, Suhail Akhtar, Stefan J. Green, Ankur Naqib, Omair Khan, Abigail R. Cannon, Adam M. Hammer, Niya L. Morris, Xiaoling Li, Joshua M. Eberhardt, Richard L Gamelli, Richard H. Kennedy, Mashkoor A. Choudhry

**Affiliations:** 1 Burn & Shock Trauma Research Institute, Loyola University Chicago Health Sciences Division, Maywood, IL, 60153, United States of America; 2 Integrative Cell Biology Program, Loyola University Chicago Health Sciences Division, Maywood, IL, 60153, United States of America; 3 Departments of Surgery, Loyola University Chicago Health Sciences Division, Maywood, IL, 60153, United States of America; 4 Departments of Microbiology and Immunology, Loyola University Chicago Health Sciences Division, Maywood, IL, 60153, United States of America; 5 DNA Services Facility, University of Illinois at Chicago, Chicago, IL, 60612, United States of America; Georgia Regents University, UNITED STATES

## Abstract

Sepsis remains one of the leading causes of death in burn patients who survive the initial insult of injury. Disruption of the intestinal epithelial barrier has been shown after burn injury; this can lead to the translocation of bacteria or their products (e.g., endotoxin) from the intestinal lumen to the circulation, thereby increasing the risk for sepsis in immunocompromised individuals. Since the maintenance of the epithelial barrier is largely dependent on the intestinal microbiota, we examined the diversity of the intestinal microbiome of severely burned patients and a controlled mouse model of burn injury. We show that burn injury induces a dramatic dysbiosis of the intestinal microbiome of both humans and mice and allows for similar overgrowths of Gram-negative aerobic bacteria. Furthermore, we show that the bacteria increasing in abundance have the potential to translocate to extra-intestinal sites. This study provides an insight into how the diversity of the intestinal microbiome changes after burn injury and some of the consequences these gut bacteria can have in the host.

## Introduction

The gastrointestinal tract contains over 100 trillion microbes, termed the microbiota, that provide numerous benefits for the host such as metabolism and *de novo* synthesis of nutrients, protection against pathogenic microbes, and immune development and function [1]. Feedback between these organisms and the immune system is necessary for establishing tolerance along mucosal surfaces and maintaining the gut epithelial barrier [2]. Dysbiosis of the healthy intestinal microbiome is associated with numerous disease states: inflammatory bowel disease (IBD), autism, obesity, rheumatoid arthritis, and diabetes [3]. In IBD, it is suggested that alterations of the healthy microbiome activate the mucosal immune response, which increases intestinal permeability and allows for the translocation of microbes or microbial products into the circulation, thereby adversely impacting the host [4].

Sepsis is the leading cause of death in patients that suffer from severe trauma. It is hypothesized that sepsis stems from bacterial infections, toxins, or metabolic products that activate pattern recognition receptors and lead to a systemic inflammatory response in immunocompromised individuals [5]. Conversely, the healthy intestinal microbiome acts as a physiological microbial barrier which keeps commensal opportunistic pathogens in check by resisting microbial colonization. Therefore, it is important to understand how this microbiome is altered following injury and the role these commensal bacteria play in potentiating gut barrier dysfunction, bacterial translocation, and ultimately sepsis after injury.

Burn injury is one of the most common forms of trauma, and in patients with severe burns, 75% of all deaths are related to sepsis or infectious complications arising from injury [6]. Following insult, there is an immediate systemic inflammatory response that spreads throughout the body and affects secondary organs [7]. In addition to the skin, there is reported inflammation in the lungs, liver, and intestines after burn [8]. In the context of the gut, previous research has shown that burn injury leads to a mesenteric vasoconstriction and produces a hypoxic environment for the gut [9]. Subsequent, reperfusion of blood to the tissue produces drastic fluctuations of oxygen levels exacerbating cell stress, cell death, and ultimately leading to a breakdown of the epithelial barrier marked by increased intestinal permeability and bacterial translocation to mesenteric lymph nodes (MLN) [10]. The translocation of bacteria from the gut to MLN has been previously shown to correlate with sepsis [11]. Furthermore, there are numerous studies which suggest that Gram-negative bacterial infections play an important role in potentiating sepsis [12,13].

Therefore, we asked whether burn injury alters the homeostatic environment of the gut which allows for changes in the intestinal microbiome that favors the overgrowth of Gram-negative aerobic bacteria. This overgrowth of gut bacteria in combination with increased intestinal permeability may allow for the translocation of these bacteria to extra-intestinal sites increasing the risk of bacterial infections and predisposing patients to sepsis.

## Materials and Methods

### Ethics Statement

#### Patient Samples

Loyola University Chicago Health Sciences Division Institutional Review Board (IRB) approved these studies and informed written consent was obtained from all subjects (burn patients and controls) except burn patients with Fecal Management System (FMS). Samples from burn patients with FMS did not require a consent as the IRB waived the need for consent from the group of patients with FMS and all patient data was de-identified prior to analysis.

Feces samples were obtained from 4 burn patients admitted to Loyola University Medical Center Maywood, IL from December 2010 to November 2011; these patients sustained 25%, 32%, 44%, and 57% total body surface area (TBSA) burns, and samples were obtained 5–17 days post injury. The median age of burn patients is 49 ± 9.7 and it ranges from 36 to 59 years. There were one female and three males among burn patients included in this study. A fecal management system routinely emplaced for burned patients, was used to collect fecal samples. Patients were selected who met the following criteria: adult male or female over 18 years of age who sustained full thickness burn injury >20% TBSA, and without pre-existing clinical infections, historical evidence of gastrointestinal diseases such as Ulcerative Colitis, Crohn’s disease, or Celiacs disease, historical evidence of gastrointestinal *Clostridium difficile* infection, no antibiotic use (other than surgical prophylaxis), without peritonitis, AIDS, immune suppressing medications, or metastasized cancer.

#### Control Group

Patients with physiologically insignificant burns, i.e. superficial burns less than 10% of total body surface area (TBSA) were designated as controls. The median age of control group population is 39.6 ± 16.84 years and it ranges from 23 to 74 years. The average surface area of control population is 4.77 ± 2.44 which ranges from 1–8% TBSA. Control population include one female and seven males. A single fecal sample was obtained from 8 control patients and used as controls for comparison to those with significant burn injury. These patients did not require the use of a FMS. These patients were also subject to the above inclusion and exclusion criteria.

#### Animals

Male C57BL/6 mice, 8–9 week old, weighing 22–25 g, were obtained from Charles River Laboratories. All experiments were conducted in accordance with the guidelines set forth by the Animal Welfare Act and were approved by the Institution Animal Care and Use Committee at the Loyola University Chicago Health Sciences Division. The identification number assigned to our animal care and use protocol is IACUC 2012067. The animals were euthanized by CO_2_ asphyxiation.

### Burn Injury Procedure

Mice were anesthetized with xylazine (80 mg/Kg) and ketamine (1.25 mg/Kg) cocktail and their dorsal surface shaved. Anesthetized mice were placed in a template exposing ~20% TBSA as calculated by the Meeh formula [14]. The mice were divided into two treatment groups, those receiving burn injuries or sham injuries. The burn group was then submerged in a water bath set to ~85°C for ~9 seconds while the sham group was submerged in a water bath set to 37°C. Following burn or sham procedures, all animals were resuscitated with 1ml of saline i.p. This procedure models a ~20% TBSA full thickness third degree burn and an ~15–20% mortality within 24–48 hours after injury. The burn injury procedure described in this proposal is widely used in many previous studies [15–17] and is performed under full anesthesia and has been histologically proven to incur a full thickness, insensate lesion [18]. The entire thickness of the dermis, including peripheral sensory endings, is destroyed [18]. The health of the mice is monitored constantly for four hours after the procedure to ensure that they wake up from the anesthesia. Mice are then returned to the animal care facility and given food and water ad libitum; and are monitored for any postoperative complications twice a day until the experiment is completed. Humane endpoints were considered based on overt signs and symptoms of sepsis (piloerection, squeaking, sensitive to touch, tearing). No animals met this criteria, therefore no animals were euthanized prior to experimental endpoints (one or three days post burn) in this study. 10/66 mice died following burn injury before they were observed to exhibit these signs of sepsis. Mice were sacrificed on days one and three following injury.

### DNA and RNA Purification

One and three days after injury, the intestines of the mice were surgically removed, opened, and luminal contents were collected from the distal 5cm of the small intestine and the whole large intestine from the cecum. RNA was purified from this region of the small and large intestine tissue using RNeasy Mini Kit in combination with DNase digestion, according to the manufacturer’s protocol (Qiagen, Valencia, CA, USA). For the human patient samples, the FMS was used to flush the bowel and collect feces from the burn patients. Control patients defecated normally and samples from this group were directly collected into sterilized cups. Genomic bacterial DNA was purified from mouse and human fecal samples using the Qiagen DNA Stool Mini Kit with an initial brief sonication step in lysis buffer ASL and a high temperature 95°C incubation step to improve bacterial cell lysis.

### Microbial Community Structure Analysis

Genomic DNA (gDNA) from the feces of the small and large intestine of mice, and human stool samples was PCR amplified and prepared for next-generation sequencing (NGS) using a modified two-step targeted amplicon sequencing approach, similar to that described previously [19,20]. Genomic DNA was initially amplified with primers 27F and 534R (17), targeting the V1-V3 variable regions of Bacterial small subunit (SSU) ribosomal RNA (rRNA) genes. The primers contained 5’ common sequence tags (known as common sequence 1 and 2, CS1 and CS2) as described previously [21]. The forward primer, CS1_27YF (ACACTGACGACATGGTTCTACA AGAGTTTGATCCTGGCTCAG) and CS2_534R (TACGGTAGCAGAGACTTGGTCT ATTACCGCGGCTGCTGG) were synthesized by Integrated DNA Technologies (IDT; Coralville, Iowa) as standard oligonucleotides. Common sequences are underlined. PCR reactions were performed according to the Human Microbiome Project (HMP) 16S 454 sequencing protocol [22], with some modifications. PCR amplifications were performed in 10 microliter reactions in 96-well plates. A mastermix for the entire plate was made using the 2X AccuPrime SuperMix II (Life Technologies, Gaithersburg, MD). The final concentration of primers was 500 nM. From 10–50 ng of genomic DNA was added to each PCR reaction. Cycling conditions were as follows: 95°C for 5 minutes, followed by 28 cycles of 95°C for 30”, 56°C for 30” and 68°C for 5’. A final, 7 minute elongation step was performed at 68°C. Reactions were verified to contain visible amplification using agarose gel electrophoresis, in addition to no visible amplification in the no-template control prior to the second stage of PCR amplification.

A second PCR amplification was performed in 10 microliter reactions in a 96-well plate to incorporate Illumina sequencing adapters and sample-specific barcodes into amplicon pools. A mastermix for the entire plate was made using the 2X AccuPrime SuperMix II. Each well received a separate primer pair, obtained from the Access Array Barcode Library for Illumina Sequencers. The final concentration of each primer concentration was 400 nM, and each well received a separate primer set with a unique 10-base barcode (Fluidigm, South San Francisco, CA; Item# 100–4876). Separate reactions with unique barcodes were included for positive control, no-template control (reaction 1) and a second no-template control reaction containing only Access Array Barcode library primers. Cycling conditions were as follows: 95°C for 5 minutes, followed by 8 cycles of 95°C for 30”, 60°C for 30” and 68°C for 30”. A final, 7 minute elongation step was performed at 68°C. PCR yield of positive and negative controls and select samples were validated with Qubit fluorometric quantitation with the Qubit 2.0 fluorometer (Life Technologies) and with size and quantification employing an Agilent TapeStation2200 device with D1000 ScreenTape (Agilent Technologies, Santa Clara, California). After assessing no amplification in the negative controls, samples were pooled in equal volume and purified using solid phase reversible immobilization (SPRI) cleanup, implemented with AMPure XP beads at a ratio of 0.6X (v:v) SPRI solution to sample. This ratio removes DNA fragments shorter than 300 bp from the pooled libraries. Final quality control was performed using TapeStation2200 and Qubit analysis, prior to dilution to 4 pM for sequencing on an Illumina MiSeq. The pool was loaded on a MiSeq v3 flow cell at a concentration of 5.5pM and sequenced in 2x300bp paired end format using a 600 cycle MiSeq v3 reagent cartridge. Library preparation was performed at the DNA services (DNAS) facility, within the Research Resources Center (RRC) at the University of Illinois at Chicago (UIC). Library sequencing was performed at the Michigan State University (MSU) Research Technology Support Facility (RTSF).

Raw sequence data were imported into the software package CLC genomics workbench (v7.0; CLC Bio, Qiagen, Boston, MA). Sequences were quality trimmed (Q20) and reads shorter than 200 bases were removed. Due to amplicon size and quality trimming, forward and reverse reads could not be consistently merged. Therefore, only the forward read was used for community analyses. The trimmed sequences were exported as FASTA files. Subsequently, FASTA files were processed through the software package QIIME. Briefly, sequences were screened for chimeras using the usearch61 algorithm [23], and putative chimeric sequences were removed from the dataset. Subsequently, each sample sequence set was sub-sampled to the smallest sample size to avoid analytical issues associated with variable library size [24]. Sub-sampled data were pooled and renamed, and clustered into operational taxonomic units (OTU) at 97% similarity. Representative sequences from each OTU were extracted, and these sequences were classified using the “assign_taxonomy” algorithm implementing the RDP classifier, with the Greengenes reference OTU build [25,26]. A biological observation matrix (BIOM; [27]) was generated at taxonomic levels from phylum to genus using the “make_OTU_table” algorithm. The BIOMs were imported into the software package Primer6 for statistical analysis and visualization using group-average clustering, non-metric multidimensional scaling (NMDS), and analysis of similarity (ANOSIM), as described previously[28,29]. Differences in the relative abundance of individual taxa between *a priori* defined groups (e.g., control and burn patients) were tested for significance using the “group_significance” algorithm, implemented within QIIME. Tests were performed using the non-parametric Kruskal-Wallis one-way analysis of variance, generating a Benjamini-Hochberg false-discovery rate (FDR) corrected p-value. Taxa with an average abundance of <1% across the entire sample set were removed from such analyses.

### Quantitative Analyses of Fecal Microbiome

Real time quantitative PCR (qPCR) was used to quantify bacterial SSU (16S) rRNA gene abundance, as described previously [30]. Primer sets targeting SSU rRNA genes of microorganisms at the domain level (*i*.*e*., Bacteria) and at the family level (*i*.*e*., Enterobacteriaceae) were used. Primers included 340F (ACTCCTACGGGAGGCAGCAGT) and 514R (ATTACCGCGGCTGCTGGC) for domain-level analyses and 515F (GTGCCAGCMGCCGCGGTAA) and 826R (GCCTCAAGGGCACAACCTCCAAG) for Enterobacteriaceae analyses. Primers were synthesized by Invitrogen. qPCR master mixes contained 1X iTaq Universal SYBR Green Supermix (Bio-rad), and 300 nM forward and reverse primers. For standards, 10-fold dilutions were made from purified genomic DNA from reference bacteria as described previously [30]. Reactions were run at 95°C for 3’, followed by 40 cycles of 95°C for 15” and a 63°C (Bacteria) or 67°C (Enterobacteriaceae) for 60”. Reactions were performed using a Step One Plus qPCR instrument (Applied Biosystems).

### Histology

Small, 3–5mm sections of tissue were taken from the ileocecal wall and fixed in Carnoy solution overnight. Paraffin blocks were prepared by the Loyola University health Sciences Division Processing Core, 5 μm sections were cut, and 1 slide from each animal was H&E stained for tissue pathology. The procedure for fluorescent in-situ hybridization staining was performed as described previously with minor adjustments [31]. Slides were deparaffinized, dried, and incubated with the indicated probes at a final concentration of 1ng/μl in hybridization buffer (0.9M NaCl, 20mMTris-HCL, pH 7.5, 0.1% SDS) and left to incubate overnight at 50°C in a dark, humidified, Tupperware container. The probe sequences were as follows and purchased from Invitrogen[30,32–35]: Universal bacterial probe EUB338: Alexa 555 5’-GCTGCCTCCCGTAGGAGT -3’ Enterobacteriaceae probe ENTBAC 183: Alexa 488 5’-CTCTTTGGTCTTGCGACG -3’ Following the incubation, the slides were washed 3x for 15min. in prewarmed wash buffer (0.9M NaCl, 20mMTris-HCL, pH 7.5,0.1% SDS) at 50°C. The slides were air dried, counterstained, and mounted using ProLong Gold Antifade Reagent with DAPI (Molecular Probes). The sections were imaged using a Zeiss Axiovert 200m fluorescent microscope and images were processed using Axiovision software.

### Intestinal Permeability

One day after the burn or sham injury procedure the mice were gavaged with 0.4 ml of 22 mg/ml FITC-dextran in PBS. After 3 hours, blood was drawn and the mice were sacrificed. The blood was centrifuged to collect the plasma, and read spectrophotometrically at 480 nm excitation and 520 nm emission wavelengths. The concentration of FITC-dextran in the plasma was determined by relating its absorbance to a standard curve of known FITC-dextran concentrations.

#### Intestinal Expression of Claudin 4, and 8

RNA from the distal small intestine tissue and large intestine was purified as described above and reverse transcribed to cDNA using High Capacity cDNA Reverse Transcription Kit (Life Technologies). Expression levels of claudin 4, and 8 were quantified by qPCR using TaqMan primer probes and Taqman Fast Advanced Master Mix (Life Technologies) and ΔCt calculations were conducted using the endogenous control gene Gapdh.

#### Cultivation of Micro-organisms

The mesenteric lymph nodes were aseptically removed, weighed, and homogenized in PBS to achieve a 50 mg/ml (MLN- wt/vol) concentration. Equal amounts of homogenate were plated on Tryptic soy agar plates with 5% sheep blood, and MacConkey agar to grow total and Gram-negative bacteria respectively. The plates were cultured aerobically in a 37°C incubator with 5% CO_2_ for 24 hours.

### Statistical Analysis

Data are expressed as mean ± standard error of the mean (SEM). Differences between groups were determined by ANOVA with Tukey’s post hoc test or Student’s t-test using GraphPad InStat. P<0.05 was considered statistically significant.

### Data Access

The amplicon sequence data from this study have been submitted to the NCBI Sequence Read Archive (SRA; http://www.ncbi.nlm.nih.gov/Traces/sra/sra.cgi) under the BioProject (PRJNA273295) accession number SRP052710. Sequences derived from mice were uploaded as two independent FASTQ files representing forward and reverse reads from each sample. In sequences derived from human feces, sequence reads were imported into the software package CLC genomics workbench and mapped against the Hg19 human genome reference. Reads mapping to the human genome (<0.05%) were removed from the dataset, and single FASTQ files, containing both forward and reverse reads were provided to the SRA.

## Results

### Burn Injury and the Structure of the Human Intestinal Microbiome

**Fig 1:**
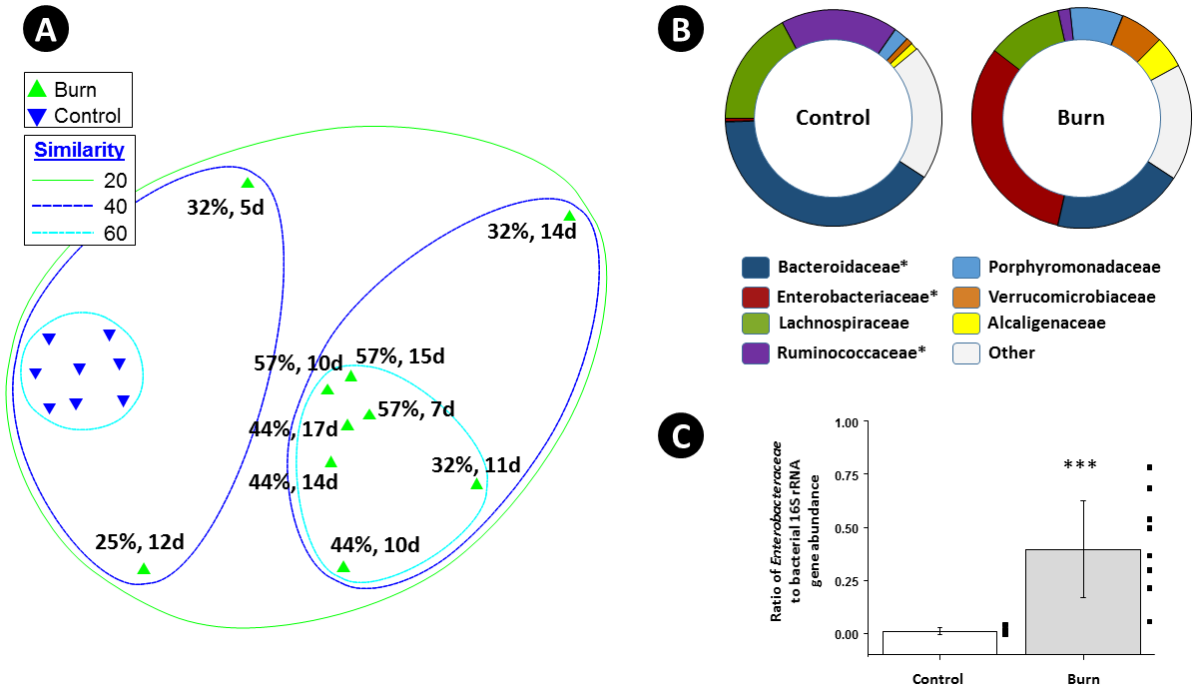
Effects of Burn Injury on Human Gut Microbiome. The non-metric multidimensional scaling (NMDS) plot (**A**) demonstrates the effect of burn injury on the overall human fecal microbial community structure, as assessed by bacterial ribosomal RNA gene amplicon sequencing. The NMDS plot is based on sample-standardized and log transformed abundance data. The NMDS plot and the hierarchical cluster overlay are based on a resemblance matrix calculated using Bray-Curtis similarity. The 2D stress value was 0.10. Fecal samples were taken from eight control patients and from four burn injury patients and analyzed at different time points (days after injury), as indicated in the figure. The most abundant bacterial families in control patients and burn patients are indicated in pie charts **(B)**, and taxa which were significantly different by Kruskal-Wallis one-way analysis of variance (*,FDR-P <0.05). A box-plot of the distribution of the ratio of rRNA genes from Enterobacteraceae to total bacterial rRNA genes in control and burn patients measured by qPCR is shown **(C)**, and the ratio of each individual is shown adjacent to the box-plot. A statistically significant effect of burn injury was observed (two-tailed t-test ***, p< 0.0001).

To examine the structure of the intestinal microbiome after burn injury, deep sequencing of bacterial SSU rRNA genes (V1-V3 region) was performed, using a PCR-NGS approach. A minimum of 40,000 raw sequences was generated per sample. After chimera removal and sub-sampling, a biological observation matrix (BIOM) was generated using 25,000 sequences per sample. The fecal microbial community structure of control and burn injury patients was analyzed, and revealed a substantial and significant effect of burn injury (Fig 1). An analysis of similarity (ANOSIM) demonstrated a significant difference between control and burn injury patients (Global R = 0.632; p = 0.2%, 999 permutations; control (N = 8 individuals and 8 total samples) and burn injury patients (N = 4 individuals and 10 total samples)). Fecal microbial community richness at the family level was significantly higher for control patients relative to burn injury patients (an average of 32.63 families vs 27.60 families; p<0.02, two-tailed TTEST, unequal variance); no other calculated indices were significantly different (*i*.*e*. Pielou’s evenness or Shannon index) (S1 and S2 Tables).

The gut microbial communities of control patients clustered together, and were divergent from all fecal samples of burn injury patients, regardless of sampling times. The two patients with the greatest TBSA had the most similar microbial community structure, regardless of sampling time, with Bray-Curtis similarity of >60% (Fig 1). Initially, the two patients with lower TBSA had distinct fecal microbial communities from those of patients with 44 and 57% TBSA. However, 11 days after injury, the fecal microbiome of the patient with 32% TBSA shifted towards those of the patients with 44% and 57% TBSA (Fig 1). The patients with 32%, 44%, and 57% TBSA died from sepsis, while the patient with 25% TBSA survived. Fecal microbial communities of control patients were dominated by bacteria from the families Bacteroidaceae, Lachnospiraceae, and Ruminococcaceae (Fig 1B), confirming earlier reports of the dominant intestinal bacteria [36–38]. The fecal microbiome of burn patients was significantly different from those of control individuals, and bacteria from the families Bacteroidaceae, Enterobacteriaceae, and Lachnospiraceae were the most abundant taxa in the fecal microbiome of burn injury patients (Fig 1B). Dramatic and significant differences in the relative abundance of these families were observed in fecal microbiome of control and burn patients (Table 1). In particular, the relative average abundance of bacteria from the family Enterobacteriaceae was higher in burn injury patients relative to control patients (average 31.9% to 0.5%). Conversely, significant decreases in the relative abundance of bacteria from the families Bacteroidaceae, and Ruminococcaceae were observed (Fig 1B; Table 1).

**Table 1:** Significant Effects of Burn Injury on Gut Microbial Community Structure.

	FDR_P	Group 1 mean	Group 2 mean
**Control vs Burn (Human)**		**Control**	**Burn**
Bacteroidetes;c__Bacteroidia;o__Bacteroidales;f__Bacteroidaceae	0.004	40.3%	19.3%
Firmicutes;c__Clostridia;o__Clostridiales;f__	0.008	4.4%	0.8%
Firmicutes;c__Clostridia;o__Clostridiales;f__Clostridiaceae	0.008	0.4%	0.1%
Firmicutes;c__Clostridia;o__Clostridiales;f__Ruminococcaceae	0.004	17.5%	1.8%
Firmicutes;c__Erysipelotrichi;o__Erysipelotrichales;f__Erysipelotrichaceae	0.007	3.3%	0.4%
Proteobacteria;c__Gammaproteobacteria;o__Enterobacteriales;f__Enterobacteriaceae	0.004	0.5%	31.9%
Unassigned	0.016	0.6%	0.4%
**Small intestine (SI) Sham vs SI_Burn-1day (Mouse)**		**SI_sham**	**SI_B1**
Bacteroidetes;c__Bacteroidia;o__Bacteroidales;f__Bacteroidaceae	0.049	0.3%	5.8%
Bacteroidetes;c__Bacteroidia;o__Bacteroidales;f__Porphyromonadaceae	0.049	0.1%	1.7%
Bacteroidetes;c__Bacteroidia;o__Bacteroidales;f__S24-7	0.045	52.0%	20.2%
Proteobacteria;c__Gammaproteobacteria;o__Enterobacteriales;f__Enterobacteriaceae	0.045	0.1%	23.8%
Unassigned	0.045	8.9%	2.4%
**Large intestine (LI) sham vs LI Burn-1day (Mouse)**		**LI_sham**	**LI_B1**
Bacteroidetes;c__Bacteroidia;o__Bacteroidales;f__Bacteroidaceae	0.034	2.9%	10.7%
Bacteroidetes;c__Bacteroidia;o__Bacteroidales;f__Porphyromonadaceae	0.034	1.5%	3.9%
Firmicutes;c__Erysipelotrichi;o__Erysipelotrichales;f__Erysipelotrichaceae	0.049	0.1%	0.8%
Proteobacteria;c__Betaproteobacteria;o__Burkholderiales;f__Alcaligenaceae	0.034	0.1%	0.3%
Proteobacteria;c__Gammaproteobacteria;o__Enterobacteriales;f__Enterobacteriaceae	0.034	0.0%	0.6%
**SI_Sham vs SI_Burn-3d (Mouse)**	No significantly differently abundant taxa		
**SI_Burn-1d vs SI_Burn-3d vs (Mouse)**	No significantly differently abundant taxa		
**LI_Sham vs LI_Burn-3d (Mouse)**	No significantly differently abundant taxa		
**LI_Burn-1d vs LI_Burn-3d (Mouse)**	No significantly differently abundant taxa		

The dramatic increase in the relative abundance of bacteria from the family Enterobacteriaceae was confirmed using quantitative PCR. Quantitative analyses of 16S rRNA genes of Enterobacteriaceae revealed a 37-fold increase in the relative abundance of Enterobacteriaceae in feces from burn injury patients relative to those from control patients (Fig 1C). Most, but not all the sequences assigned to the family Enterobacteriaceae could not be classified to the level of genus; however, bacteria from the genera *Citrobacter*, *Enterobacter*, *Erwinia*, *Escherichia*, *Klebsiella*, *Proteus*, *Serratia*, *and Trabulsiella* were detected. The most abundant taxon (OTU) detected in burn patients had a 16S rRNA gene sequence that was highly similar (>99.5%; 279/280 matching bases) to that of the adherent invasive *E*. *coli* strain O83:H1. The representative gene sequence of this taxon was 100% identical to a number of strains of bacteria from the genera *Enterobacter* and *Escherichia*. This single taxon represented nearly 60% of all Enterobacteriaceae sequences recovered in all samples.

### The Effect of Burn Injury on the Mouse Intestinal Microbiome

**Fig 2:**
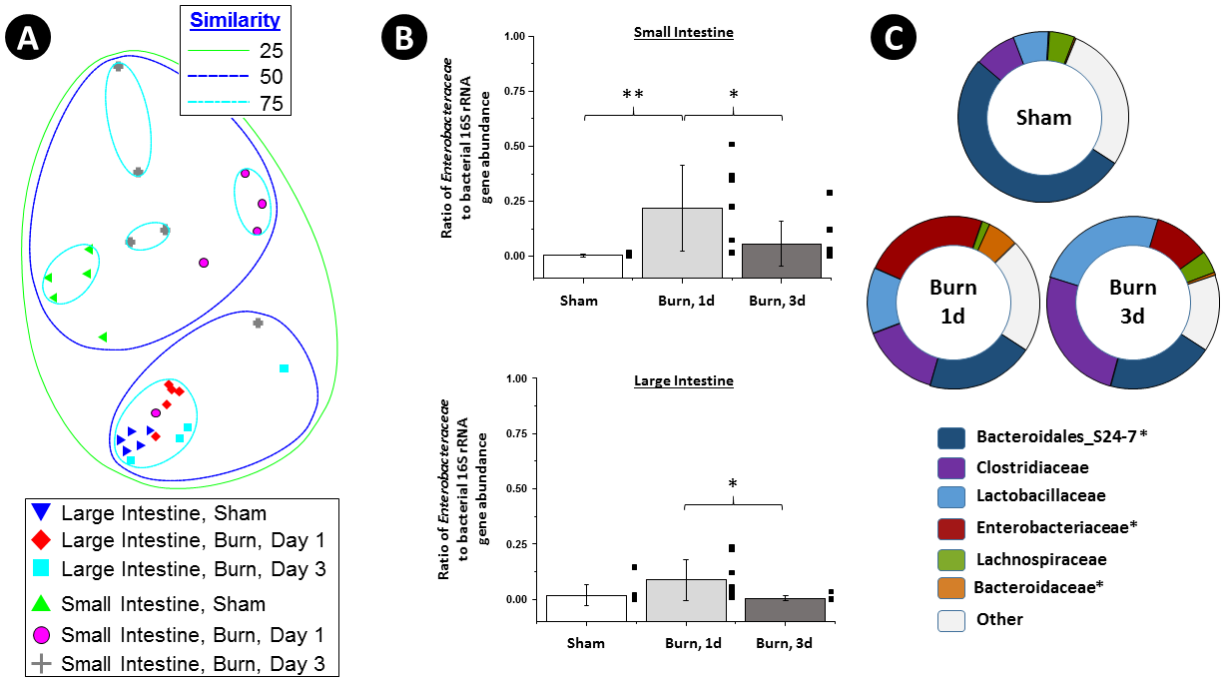
Burn Injury Alters the Mouse Intestinal Microbiome. The non-metric multidimensional scaling (NMDS) plot (**A**) demonstrates the differences in microbial community structure as a result of burn injury, time since burn injury, and sampling from small and large intestine in a mouse experimental model system of five animals per group. Box-plots of the distribution of the ratio of rRNA genes from Enterobacteriaceae to total bacterial rRNA genes in sham and burn animals, calculated by qPCR, are shown for small and large intestines, one and three days post-burn injury **(B)**. A statistically significant effect of burn injury was observed between sham and one-day burn injury mice in the small intestine (n = 9 sham, 7 burn mice, two-tailed t-test **, p< 0.01), and between one-day and three-day burn injury mice (n = 9 sham, 8 burn mice, two-tailed t-test *, p< 0.05). A statistically significant effect of burn injury was observed for the large intestine between one-day and three-day burn injury mice (n = 8 animals per group, two-tailed t-test *, p< 0.05). The most abundant bacterial families in sham and burn injury mice (small intestine) are indicated in pie charts **(C)**, and taxa which were significantly different by Kruskal-Wallis one-way analysis of variance (*, FDR-P <0.05).

The effect of burn injury on the gut microbial community was examined in a mouse model experimental system. These studies were performed to determine if (a) the shift in gut microbial community structure in human patients was reproducible in mice; (b) determine if similar microorganisms developed in the gut of burn injury mice as in humans; and (c) determine if differences in community structure were observed in multiple locations in the gastrointestinal tract. Microbial community structure was assessed in the large and small intestines of mice, one and three days after burn or sham burn treatment. Genomic DNA extracts were processed as described for human fecal samples, and a biological observation matrix (BIOM) was generated using 25,000 sequences per sample (Fig 2). Significant differences in microbial community structure between large and small intestine were observed, independent of treatment or date (ANOSIM, Global R = 0.619, p<0.002, 999 permutations). The effect of burn injury on the microbial community structure in the large intestine was smaller than that in the small intestine. Nonetheless, a moderate, but not significant shift, was observed in large intestine samples (ANOSIM, Global R = 0.218, p = 0.059, 999 permutations) across all time points. When samples from only the first day post-burn were considered, a significant effect was observed (ANOSIM, Global R = 0.872, p = 0.008, 126 permutations). A similar effect was observed in the small intestine samples (ANOSIM, Global R = 0.265, p = 0.02, 999 permutations), particularly when only sham and day 1 samples were compared (Global R = 0.672, p = 0.008, 126 permutations). No significant differences in any calculated diversity index was observed between the small intestine microbiomes of no burn injury (sham) and burn injury mice (S1 and S2 Tables). In the large intestine microbiome, the evenness and diversity of the burn injury mice at 1 day was slightly, but significantly, different than that of sham mice or burn injury mice at 3 days (*e*.*g*. Shannon index of 2.32 vs 2.13 or 2.10; p<0.004; two-tailed TTEST, unequal variance; S1 and S2 Tables).

The relative abundance of bacteria from the family Enterobacteriaceae in the gut of mice experiencing burn injury substantially increased one day after burn injury, relative to the sham control, and on average decreased three days after burn injury (Fig 2). The effect was significant in the microbial communities from the small intestine after one day (Fig 2; Table 1). In the small and large intestine, the relative abundance of Enterobacteriaceae decreased significantly from day one to day three, but was not significantly different at day three from the sham (Fig 2; Table 1). In addition, the relative abundance of other microbial families was significantly altered between treatments and time points. For example, in the analysis of small intestine microbial communities, the average relative abundance of SSU rRNA genes of bacteria from the “S24-7” group of the Bacteroidales and bacteria from the family Bacteroidaceae was significantly lower in burn injury mice at one day (Fig 2; Table 1).

The effect of burn injury on the microbial community in the large intestine was different than that observed for small intestine (Fig 2). The abundance of bacteria from the family Enterobacteriaceae was generally much lower in the large intestine than in the small intestine (on average, less than 1% of all bacterial sequences), regardless of condition (Table 1). Nonetheless, shifts in the relative abundance of bacteria from the family Enterobacteriaceae were observed, and the effect was significant by sequence analysis, though not by qPCR (Fig 2; Table 1). The average relative abundance of bacteria from the family Bacteroidaceae, Porphyromonadaceae, Erysipelotrichaceae, and Alcaligenaceae were all significantly higher in burn injury mice at day 1, though these taxa were of moderate or low overall relative abundance in the large intestine microbiome (Table 1). Abundant taxa in the large intestine, such as the “S24-7” group, and the families Lachnospiraceae, Prevotellaceae, Rikenellaceae, and Ruminococcaceae were not significantly differently abundant in the between sham and burn injury mice.

### Bacterial Translocation of Enterobacteriaceae

**Fig 3:**
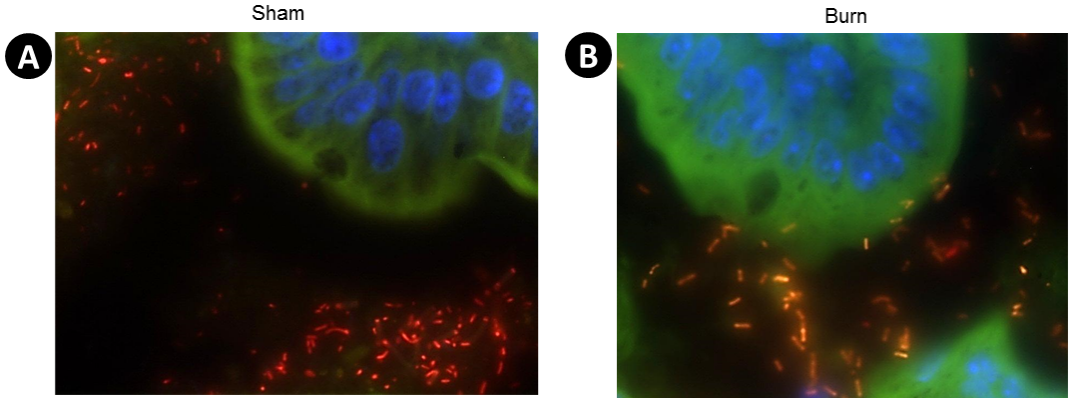
Direct Observation of Enterobacteriaceae in the Small Intestine. Tissue sections were taken from the small intestine of sham or burn injured animals one day after injury, and stained with fluorescently labeled oligonucleotide conjugated probes to label 16s rRNA and counterstained with DAPI to label intestinal nuclei blue. Alexa 555 (Red) EUB 338 probe was used to stain total Bacteria, and Alexa 488 (Green) ENTBAC 183 was used to stain Enterobacteriaceae. Orange depicts co-localization of both probes and bacteria that are Enterobacteriaceae. (**A**) is a representative picture of sham animals and (**B**) is a representative image of burn injury animals.

Bacteria were identified in the small intestine using fluorescence *in-situ* hybridization (FISH) analysis, employing family-level (Enterobacteriaceae) and domain-level (Bacteria) oligonucleotide probes targeting the SSU rRNAs. These analyses were used to visualize the proximity of bacteria to the small intestinal villi. In the sham mice, Enterobacteriaceae were present in low relative abundance and were rarely attached to the intestinal villi (Fig 3). After burn injury, bacteria from the family Enterobacteriaceae were observed adhering to or adjacent to the small intestinal villi (Fig 3B).

**Fig 4:**
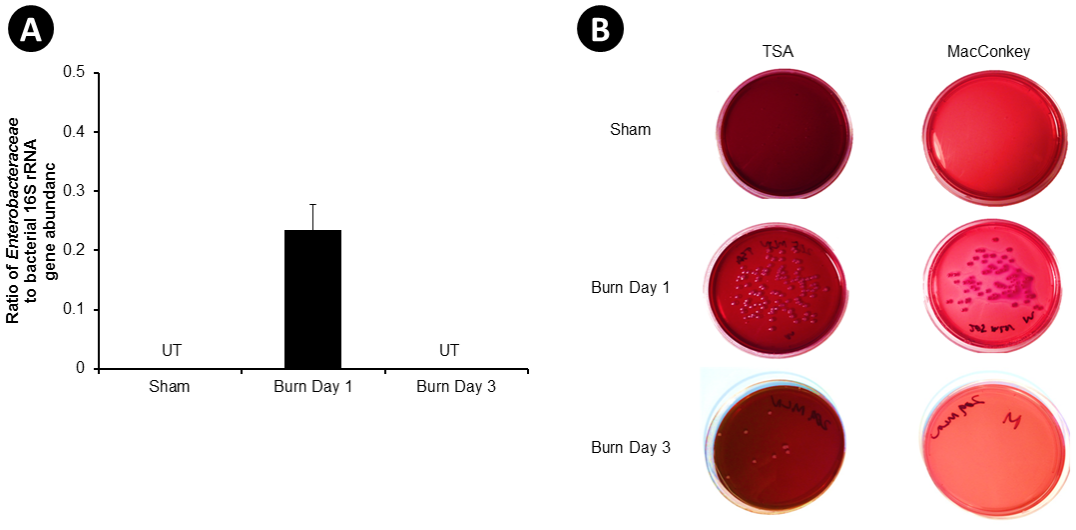
Bacterial Abundance in the Mesenteric Lymph Nodes (MLN) of Mice. MLN were aseptically removed from sham, burn day 1, and burn day 3 animals and Enterobacteriaceae were quantified by qPCR and standardized by total bacterial 16s rRNA gene abundance, (**A**). Data were expressed as mean ± SEM of 5–11 animals per group. Enterobacteriaceae abundance from sham and burn day 3 animals were all not detectable (ND). In addition, MLN homogenates were plated on Tryptic Soy Agar with 5% sheep blood and MacConkey Agar plates and cultured aerobically for 24 hours with 5% CO_2._ (**B**) is a representative image of plates produced from one animal.

The abundance of Enterobacteriaceae in the MLN was measured using qPCR of genomic DNA extracted from the MLN, and through bacterial cultivation. qPCR analyses detected Enterobacteriaceae one day after injury in the MLN (Fig 4A). To determine if these bacteria were viable, MLN homogenates were cultured aerobically for 24 hours on Tryptic Soy Agar (TSA) with blood to identify total aerobic bacteria, and on MacConkey Agar to identify Gram-negative aerobic bacteria, including Enterobacteriaceae. Colonies were observed to develop on TSA and MacConkey plates in all burn injured animals one day after injury, while no colonies were observed on the plates inoculated with homogenate from sham animals (Fig 4B). Three days after burn, some colonies were detected on the TSA plates, but no colonies were detected on the MacConkey agar.

### Burn Injury Increases Intestinal Permeability

**Fig 5:**
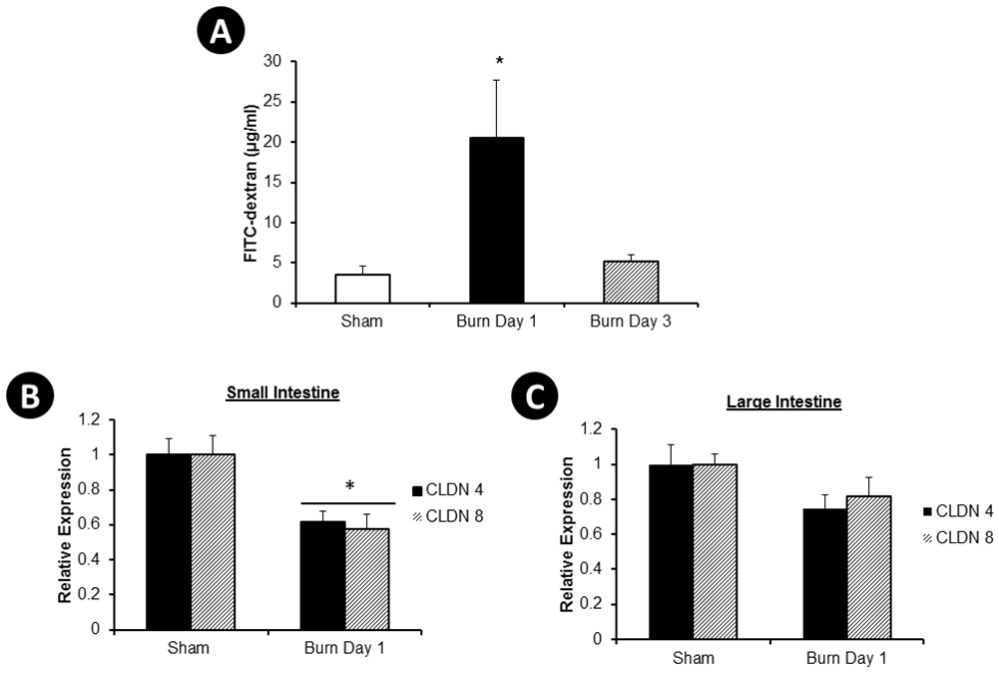
Intestinal Permeability. Sham, burn day 1, and burn day 3 mice were gavaged with FITC-dextran and 3 hours later blood was drawn and the concentration of FITC-dextran was determined spectrophotometrically in the plasma (**A**). RNA was purified from the distal small (**B**) and large (**C**) intestine one day after burn, reverse transcribed and quantified with qPCR using primers for claudin (Cldn 4, and Cldn 8), in combination with endogenous control Gapdh. ΔCt values were calculated and the mean ± SEM of 10–15 animals/group was expressed relative to sham. (**A**), *, p<0.05 ANOVA followed by Tukey-Kramer multiple comparisons post hoc test of sham and burn day 1. (**B**), unpaired student t-test sham and burn day 1, * p<0.05.

Increased gut leakiness can result in bacterial translocation from the gut to the lymph nodes. Intestinal permeability was measured *in vivo* one and three days after burn with a FITC-dextran permeability assay. Sham and burn injured mice were gavaged with FITC-dextran one and three days after burn. Three hours later, the concentration of this dye was determined spectrophotometrically in the plasma. An increase in the concentration of FITC-dextran was observed in mice one day after burn, and no change was observed three days after injury relative to the sham animals (Fig 5A). In addition, gene expression of two tight junction proteins, claudin 4, and 8 were measured in the small and large intestine of sham and burn injury mice. Gene expression levels of claudin 4 and 8 decreased by ~40% in the small intestine one day after injury (Fig 5B). A smaller, and not significant, change was observed in the large intestine (Fig 5C).

## Discussion

In this study, we show that burn injury alters the structure of the intestinal microbiome promoting the overgrowth of specific Gram-negative aerobic bacteria, but within the context of fairly limited effects on overall microbial diversity. The overgrowth of Enterobacteriaceae coupled with the increase of intestinal permeability seen one day after burn allows for the translocation of these bacteria to the mesenteric lymph nodes. This provides evidence that the gut may be a source of bacterial infections after burn injury, and a potential cause of sepsis.

Examining the structure of the intestinal microbiome of severely burned patients, we found that injury promotes the overgrowth of many under representative taxa while reducing the overall healthy diversity of bacteria. This shift in the microbiome is similarly seen in other inflammatory conditions, such as IBD [39], and consequently may also yield profound implications for treatment of infection and immune modulation in trauma patients. The most profound changes in the microbiome were dramatic increases in the abundance of γ-Proteobacteria, particularly those within the family Enterobacteriaceae. This family contains many opportunistic pathogenic bacteria, including those from the genera *Escherichia*, *Klebsiella*, *Proteus*, *and Citrobacter*, which are common in septic patients [11]. Bacteria from the family Enterobacteriaceae are potentially proinflammatory and have been shown to induce spontaneous colitis when transferred to wild type mice [34]. More research is needed to determine which strains of these bacteria elicit systemic inflammation after burn injury. Additional sequencing efforts, including assembly of full length SSU rRNA gene amplicons, and deep shotgun metagenome sequencing, will be instrumental in more accurately identifying the burn injury-associated Enterobacteriaceae, and in determining specific physiological capabilities enabling their dramatic overgrowth after burn injury.

In addition to overgrowth of potentially pathogenic bacteria, we observed reductions in potentially protective bacteria. The Lachnospiraceae are a Gram-positive family of bacteria within the phylum Firmicutes, and include bacteria from the genus *Clostridium*. Various species of spore forming bacteria under this cluster have been shown to ferment carbohydrates to produce butyrate, induce Treg induction, and prevent inflammation in models of colitis [40–43]. Reductions of this family of bacteria have been observed in IBD, and it is possible that these species of bacteria are also protective in maintaining the gut barrier integrity after trauma [39,44]. If so, reconstitution of these strains through probiotic supplementation may prove to be a novel treatment to burn patients, ideally leading to reduced bacterial translocation and sepsis.

More research is needed to identify the cause of the dramatic shifts in bacterial community structure associated with burn injury. Two potential mechanisms are increased intestinal inflammation and reductions of antimicrobial peptides. Previous research has shown that intestinal inflammation alters the intestinal microbiome, and allows for an overgrowth of Enterobacteriaceae. Bacteria from the family Enterobacteriaceae have been shown to outcompete other resident bacteria and reduce total bacterial numbers [45]. Another study demonstrated that host generated nitrate produced as a by-product of the inflammatory response can lead to boosts of E.coli in the inflamed gut [46]. In addition, α-defensins and C-type lectins, two classes of host-produced antimicrobial peptides, have been implicated in the establishment and regulation of the intestinal microbiota. Recent studies have shown that a reduction in α-defensins promote shifts in microbial communities, leading to the overgrowth of pathogenic bacteria and intestinal inflammation in Crohn’s disease [32,47,48]. C-type lectins are another class of antimicrobials which protect against intestinal inflammation and colitis by segregating the commensal bacteria from the intestinal epithelium [49]. Therefore a potential decrease in these antimicrobials may help explain the shifts in bacterial abundance.

Our findings further demonstrate that burn injury leads to an increase in gut leakiness which allows for bacterial translocation to the MLN. Tight junction (TJ) proteins, such as claudins are indispensable in maintaining the permeability of the intestine. Diseases where TJ protein expression is altered have been shown to correlate with the translocation of bacterial products to the circulation [50,51]. There was a significant decrease in claudin 4, and 8, which accompanied the increase in Enterobacteriaceae seen in the small intestine following burn injury. Reports have shown various Proteobacteria with the potential to modulate claudin 4 expression and permeability in the intestine[52,53]. Reduced claudin 8 expression has been observed in diseases of intestinal barrier dysfunction such as Crohn’s disease and in autism models where dysbiosis is also evident[50,51]. There seems to be a mutual relationship between dysbiosis of the microbiome and altered TJ proteins. However, it is not well established whether dysbiosis precedes and causes alterations in intestinal permeability, or whether altered permeability can directly change the microbiome.

To our knowledge this is the first study that investigates the structure of the intestinal microbiome in severely burned patients. The relatively few patient samples, their individual antibiotic regimens, and when the fecal management system was utilized in the clinical care of the patients are all confounding factors to this study. Nevertheless, comparison of the burn patients’ intestinal microbiome with that of our mouse model revealed many similar trends providing strong evidence that trauma modifies the intestinal homeostatic environment, thereby resulting in alterations in the intestinal microbiome, and overgrowth of Enterobacteriaceae. Translocation of Enterobacteriaceae to the MLN and systemic Gram-negative bacteremia can lead to sepsis and multiple organ failure for burn patients.

## Supporting Information

S1 Table Alpha diversity calculations based on microbial 16S rRNA gene amplicon sequence data. (DOCX)

S2 Table Group differences in Alpha diversity indices. (DOCX)

## References

[1] Round JL, Mazmanian SK. The gut microbiota shapes intestinal immune responses during health and disease. Nat Rev Immunol. 2009;9: 313–323. 10.1038/nri2515 19343057 PMC4095778

[2] Honda K, Littman DR. The microbiome in infectious disease and inflammation. Annu Rev Immunol. 2012;30: 759–795. 10.1146/annurev-immunol-020711-074937 22224764 PMC4426968

[3] Clemente JC, Ursell LK, Parfrey LW, Knight R. The impact of the gut microbiota on human health: An integrative view. Cell. 2012;148: 1258–1270. 10.1016/j.cell.2012.01.035 22424233 PMC5050011

[4] Xavier RJ, Podolsky DK. Unravelling the pathogenesis of inflammatory bowel disease. Nature. 2007;448: 427–434. 17653185 10.1038/nature06005

[5] Murphy TJ, Paterson HM, Mannick JA, Lederer JA. Injury, sepsis, and the regulation of toll-like receptor responses. J Leukoc Biol. 2004;75: 400–407. 14557385 10.1189/jlb.0503233

[6] Church D, Elsayed S, Reid O, Winston B, Lindsay R. Burn wound infections. Clin Microbiol Rev. 2006;19: 403–434. 16614255 10.1128/CMR.19.2.403-434.2006 PMC1471990

[7] Stoecklein VM, Osuka A, Lederer JA. Trauma equals danger--damage control by the immune system. J Leukoc Biol. 2012;92: 539–551. 10.1189/jlb.0212072 22654121 PMC3427603

[8] Shankar R, Melstrom KA Jr, Gamelli RL. Inflammation and sepsis: Past, present, and the future. J Burn Care Res. 2007;28: 566–571. 17665516 10.1097/BCR.0B013E318093DF16

[9] Magnotti LJ, Deitch EA. Burns, bacterial translocation, gut barrier function, and failure. J Burn Care Rehabil. 2005;26: 383–391. 16151282 10.1097/01.bcr.0000176878.79267.e8

[10] Choudhry MA, Rana SN, Kavanaugh MJ, Kovacs EJ, Gamelli RL, Sayeed MM. Impaired intestinal immunity and barrier function: A cause for enhanced bacterial translocation in alcohol intoxication and burn injury. Alcohol. 2004;33: 199–208. 15596088 10.1016/j.alcohol.2004.05.004

[11] MacFie J, O'Boyle C, Mitchell CJ, Buckley PM, Johnstone D, Sudworth P. Gut origin of sepsis: A prospective study investigating associations between bacterial translocation, gastric microflora, and septic morbidity. Gut. 1999;45: 223–228. 10403734 10.1136/gut.45.2.223 PMC1727620

[12] Poltorak A, He X, Smirnova I, Liu MY, Van Huffel C, Du X, et al Defective LPS signaling in C3H/HeJ and C57BL/10ScCr mice: Mutations in Tlr4 gene. Science. 1998;282: 2085–2088. 9851930 10.1126/science.282.5396.2085

[13] Levin J, Poore TE, Zauber NP, Oser RS. Detection of endotoxin in the blood of patients with sepsis due to gran-negative bacteria. N Engl J Med. 1970;283: 1313–1316. 5478453 10.1056/NEJM197012102832404

[14] Gilpin DA. Calculation of a new meeh constant and experimental determination of burn size. Burns. 1996;22: 607–611. 8982538 10.1016/s0305-4179(96)00064-2

[15] Wade CE, Baer LA, Wu X, Silliman DT, Walters TJ, Wolf SE. Severe burn and disuse in the rat independently adversely impact body composition and adipokines. Crit Care. 2013;17: R225 10.1186/cc13048 24099533 PMC4057079

[16] Song J, Wolf SE, Herndon DN, Wu XW, Jeschke MG. Second hit post burn increased proximal gut mucosa epithelial cells damage. Shock. 2008;30: 184–188. 10.1097/SHK.0b013e318162a3f6 18197149 PMC7859870

[17] Song J, de Libero J, Wolf SE. Hepatic autophagy after severe burn in response to endoplasmic reticulum stress. J Surg Res. 2014;187: 128–133. 10.1016/j.jss.2013.09.042 24209807 PMC4169053

[18] Faunce DE, Llanas JN, Patel PJ, Gregory MS, Duffner LA, Kovacs EJ. Neutrophil chemokine production in the skin following scald injury. Burns. 1999;25: 403–410. 10439148 10.1016/s0305-4179(99)00014-5

[19] Bybee SM, Bracken-Grissom H, Haynes BD, Hermansen RA, Byers RL, Clement MJ, et al Targeted amplicon sequencing (TAS): A scalable next-gen approach to multilocus, multitaxa phylogenetics. Genome Biol Evol. 2011;3: 1312–1323. 10.1093/gbe/evr106 22002916 PMC3236605

[20] de Carcer DA, Denman SE, McSweeney C, Morrison M. Strategy for modular tagged high-throughput amplicon sequencing. Appl Environ Microbiol. 2011;77: 6310–6312. 10.1128/AEM.05146-11 21764953 PMC3165417

[21] Moonsamy PV, Williams T, Bonella P, Holcomb CL, Hoglund BN, Hillman G, et al High throughput HLA genotyping using 454 sequencing and the fluidigm access array system for simplified amplicon library preparation. Tissue Antigens. 2013;81: 141–149. 10.1111/tan.12071 23398507

[22] Human Microbiome Project Consortium. A framework for human microbiome research. Nature. 2012;486: 215–221. 10.1038/nature11209 22699610 PMC3377744

[23] Edgar RC. Search and clustering orders of magnitude faster than BLAST. Bioinformatics. 2010;26: 2460–2461. 10.1093/bioinformatics/btq461 20709691

[24] Gihring TM, Green SJ, Schadt CW. Massively parallel rRNA gene sequencing exacerbates the potential for biased community diversity comparisons due to variable library sizes. Environ Microbiol. 2012;14: 285–290. 10.1111/j.1462-2920.2011.02550.x 21923700

[25] Wang Q, Garrity GM, Tiedje JM, Cole JR. Naive bayesian classifier for rapid assignment of rRNA sequences into the new bacterial taxonomy. Appl Environ Microbiol. 2007;73: 5261–5267. 17586664 10.1128/AEM.00062-07 PMC1950982

[26] McDonald D, Price MN, Goodrich J, Nawrocki EP, DeSantis TZ, Probst A, et al An improved greengenes taxonomy with explicit ranks for ecological and evolutionary analyses of bacteria and archaea. ISME J. 2012;6: 610–618. 10.1038/ismej.2011.139 22134646 PMC3280142

[27] McDonald D, Clemente JC, Kuczynski J, Rideout JR, Stombaugh J, Wendel D, et al The biological observation matrix (BIOM) format or: How I learned to stop worrying and love the ome-ome. Gigascience. 2012;1: 7-217X-1-7. 10.1186/2047-217X-1-15 23587224 PMC3626512

[28] Clarke KR. Non-parametric multivariate analyses of changes in community structure. Australian Journal of Ecology. 1993;18: 117–143.

[29] Voigt RM, Forsyth CB, Green SJ, Mutlu E, Engen P, Vitaterna MH, et al Circadian disorganization alters intestinal microbiota. PLoS One. 2014;9: e97500 10.1371/journal.pone.0097500 24848969 PMC4029760

[30] Barman M, Unold D, Shifley K, Amir E, Hung K, Bos N, et al Enteric salmonellosis disrupts the microbial ecology of the murine gastrointestinal tract. Infect Immun. 2008;76: 907–915. 18160481 10.1128/IAI.01432-07 PMC2258829

[31] Canny G, Swidsinski A, McCormick BA. Interactions of intestinal epithelial cells with bacteria and immune cells: Methods to characterize microflora and functional consequences. Methods Mol Biol. 2006;341: 17–35. 16799186 10.1385/1-59745-113-4:17

[32] Salzman NH, Hung K, Haribhai D, Chu H, Karlsson-Sjoberg J, Amir E, et al Enteric defensins are essential regulators of intestinal microbial ecology. Nat Immunol. 2010;11: 76–83. 10.1038/ni.1825 19855381 PMC2795796

[33] Raetz M, Hwang SH, Wilhelm CL, Kirkland D, Benson A, Sturge CR, et al Parasite-induced TH1 cells and intestinal dysbiosis cooperate in IFN-gamma-dependent elimination of paneth cells. Nat Immunol. 2013;14: 136–142. 10.1038/ni.2508 23263554 PMC3552073

[34] Garrett WS, Gallini CA, Yatsunenko T, Michaud M, DuBois A, Delaney ML, et al Enterobacteriaceae act in concert with the gut microbiota to induce spontaneous and maternally transmitted colitis. Cell Host Microbe. 2010;8: 292–300. 10.1016/j.chom.2010.08.004 20833380 PMC2952357

[35] Loy A, Maixner F, Wagner M, Horn M. probeBase--an online resource for rRNA-targeted oligonucleotide probes: New features 2007. Nucleic Acids Res. 2007;35: D800–4. 17099228 10.1093/nar/gkl856 PMC1669758

[36] Gill SR, Pop M, Deboy RT, Eckburg PB, Turnbaugh PJ, Samuel BS, et al Metagenomic analysis of the human distal gut microbiome. Science. 2006;312: 1355–1359. 16741115 10.1126/science.1124234 PMC3027896

[37] Eckburg PB, Bik EM, Bernstein CN, Purdom E, Dethlefsen L, Sargent M, et al Diversity of the human intestinal microbial flora. Science. 2005;308: 1635–1638. 15831718 10.1126/science.1110591 PMC1395357

[38] Arumugam M, Raes J, Pelletier E, Le Paslier D, Yamada T, Mende DR, et al Enterotypes of the human gut microbiome. Nature. 2011;473: 174–180. 10.1038/nature09944 21508958 PMC3728647

[39] Frank DN, Amand AL St, Feldman RA, Boedeker EC, Harpaz N, Pace NR. Molecular-phylogenetic characterization of microbial community imbalances in human inflammatory bowel diseases. Proc Natl Acad Sci U S A. 2007;104: 13780–13785. 17699621 10.1073/pnas.0706625104 PMC1959459

[40] Atarashi K, Tanoue T, Oshima K, Suda W, Nagano Y, Nishikawa H, et al Treg induction by a rationally selected mixture of clostridia strains from the human microbiota. Nature. 2013;500: 232–236. 10.1038/nature12331 23842501

[41] Atarashi K, Tanoue T, Shima T, Imaoka A, Kuwahara T, Momose Y, et al Induction of colonic regulatory T cells by indigenous clostridium species. Science. 2011;331: 337–341. 10.1126/science.1198469 21205640 PMC3969237

[42] Ivanov II, Honda K. Intestinal commensal microbes as immune modulators. Cell Host Microbe. 2012;12: 496–508. 10.1016/j.chom.2012.09.009 23084918 PMC3516493

[43] Arpaia N, Campbell C, Fan X, Dikiy S, van der Veeken J, deRoos P, et al Metabolites produced by commensal bacteria promote peripheral regulatory T-cell generation. Nature. 2013;504: 451–455. 10.1038/nature12726 24226773 PMC3869884

[44] Sokol H, Seksik P, Furet JP, Firmesse O, Nion-Larmurier I, Beaugerie L, et al Low counts of faecalibacterium prausnitzii in colitis microbiota. Inflamm Bowel Dis. 2009;15: 1183–1189. 10.1002/ibd.20903 19235886

[45] Lupp C, Robertson ML, Wickham ME, Sekirov I, Champion OL, Gaynor EC, et al Host-mediated inflammation disrupts the intestinal microbiota and promotes the overgrowth of enterobacteriaceae. Cell Host Microbe. 2007;2: 119–129. 18005726 10.1016/j.chom.2007.06.010

[46] Winter SE, Winter MG, Xavier MN, Thiennimitr P, Poon V, Keestra AM, et al Host-derived nitrate boosts growth of E. coli in the inflamed gut. Science. 2013;339: 708–711. 10.1126/science.1232467 23393266 PMC4004111

[47] Wehkamp J, Harder J, Weichenthal M, Schwab M, Schaffeler E, Schlee M, et al NOD2 (CARD15) mutations in crohn's disease are associated with diminished mucosal alpha-defensin expression. Gut. 2004;53: 1658–1664. 15479689 10.1136/gut.2003.032805 PMC1774270

[48] Wehkamp J, Salzman NH, Porter E, Nuding S, Weichenthal M, Petras RE, et al Reduced paneth cell alpha-defensins in ileal crohn's disease. Proc Natl Acad Sci U S A. 2005;102: 18129–18134. 16330776 10.1073/pnas.0505256102 PMC1306791

[49] Vaishnava S, Yamamoto M, Severson KM, Ruhn KA, Yu X, Koren O, et al The antibacterial lectin RegIIIgamma promotes the spatial segregation of microbiota and host in the intestine. Science. 2011;334: 255–258. 10.1126/science.1209791 21998396 PMC3321924

[50] Hsiao EY, McBride SW, Hsien S, Sharon G, Hyde ER, McCue T, et al Microbiota modulate behavioral and physiological abnormalities associated with neurodevelopmental disorders. Cell. 2013;155: 1451–1463. 10.1016/j.cell.2013.11.024 24315484 PMC3897394

[51] Zeissig S, Burgel N, Gunzel D, Richter J, Mankertz J, Wahnschaffe U, et al Changes in expression and distribution of claudin 2, 5 and 8 lead to discontinuous tight junctions and barrier dysfunction in active crohn's disease. Gut. 2007;56: 61–72. 16822808 10.1136/gut.2006.094375 PMC1856677

[52] Fedwick JP, Lapointe TK, Meddings JB, Sherman PM, Buret AG. Helicobacter pylori activates myosin light-chain kinase to disrupt claudin-4 and claudin-5 and increase epithelial permeability. Infect Immun. 2005;73: 7844–7852. 16299274 10.1128/IAI.73.12.7844-7852.2005 PMC1307049

[53] Lamb-Rosteski JM, Kalischuk LD, Inglis GD, Buret AG. Epidermal growth factor inhibits campylobacter jejuni-induced claudin-4 disruption, loss of epithelial barrier function, and escherichia coli translocation. Infect Immun. 2008;76: 3390–3398. 10.1128/IAI.01698-07 18490463 PMC2493239

