# Supplementary material for: Burn Injury Alters the Intestinal Microbiome and Increases Gut Permeability and Bacterial Translocation
Source: PLoS One. 2015 Jul 8;10(7):e0129996. doi: 10.1371/journal.pone.0129996 (PMC4496078; doi:10.1371/journal.pone.0129996)
Supplement: S1 Table — (DOCX) [file pone.0129996.s001.docx]

| **Supplemental Table 1: Alpha diversity calculations based on microbial 16S rRNA gene amplicon sequence data** | | | | | | | | | | |
| --- | --- | --- | --- | --- | --- | --- | --- | --- | --- | --- |
|  |  |  |  |  | Diversity Indices* | | | | |  |
| Sample Name | Host | Source | Detail | Sampling time | S | d | J' | H'(loge) | 1-Lambda' |  |
| B1 | Human | Feces | Burn |  | 33 | 3.16 | 0.55 | 1.92 | 0.78 |  |
| B10 | Human | Feces | Burn |  | 27 | 2.57 | 0.58 | 1.92 | 0.82 |  |
| B2 | Human | Feces | Burn |  | 33 | 3.16 | 0.66 | 2.29 | 0.87 |  |
| B3 | Human | Feces | Burn |  | 26 | 2.47 | 0.34 | 1.11 | 0.62 |  |
| B4 | Human | Feces | Burn |  | 25 | 2.37 | 0.36 | 1.15 | 0.60 |  |
| B5 | Human | Feces | Burn |  | 27 | 2.57 | 0.57 | 1.87 | 0.79 |  |
| B6 | Human | Feces | Burn |  | 27 | 2.57 | 0.53 | 1.74 | 0.78 |  |
| B7 | Human | Feces | Burn |  | 27 | 2.57 | 0.38 | 1.25 | 0.63 |  |
| B8 | Human | Feces | Burn |  | 28 | 2.67 | 0.49 | 1.64 | 0.77 |  |
| B9 | Human | Feces | Burn |  | 23 | 2.17 | 0.58 | 1.83 | 0.80 |  |
| C1 | Human | Feces | Control |  | 33 | 3.16 | 0.52 | 1.83 | 0.74 |  |
| C2 | Human | Feces | Control |  | 33 | 3.16 | 0.49 | 1.72 | 0.75 |  |
| C3 | Human | Feces | Control |  | 28 | 2.67 | 0.51 | 1.70 | 0.71 |  |
| C4 | Human | Feces | Control |  | 38 | 3.65 | 0.52 | 1.88 | 0.77 |  |
| C5 | Human | Feces | Control |  | 38 | 3.65 | 0.55 | 2.02 | 0.79 |  |
| C6 | Human | Feces | Control |  | 30 | 2.86 | 0.54 | 1.82 | 0.73 |  |
| C7 | Human | Feces | Control |  | 33 | 3.16 | 0.59 | 2.08 | 0.80 |  |
| C8 | Human | Feces | Control |  | 28 | 2.67 | 0.52 | 1.75 | 0.75 |  |
| 154_L | Mouse | Large Intestine | Burn | 1D | 31 | 2.96 | 0.68 | 2.32 | 0.88 |  |
| 155_L | Mouse | Large Intestine | Burn | 1D | 34 | 3.26 | 0.69 | 2.43 | 0.89 |  |
| 157_L | Mouse | Large Intestine | Burn | 1D | 33 | 3.16 | 0.67 | 2.34 | 0.88 |  |
| 158_L | Mouse | Large Intestine | Burn | 1D | 29 | 2.77 | 0.65 | 2.19 | 0.86 |  |
| 159_L | Mouse | Large Intestine | Burn | 1D | 31 | 2.96 | 0.68 | 2.33 | 0.88 |  |
| 171_L | Mouse | Large Intestine | Burn | 3D | 29 | 2.77 | 0.61 | 2.04 | 0.80 |  |
| 172_L | Mouse | Large Intestine | Burn | 3D | 30 | 2.86 | 0.59 | 2.01 | 0.81 |  |
| 173_L | Mouse | Large Intestine | Burn | 3D | 30 | 2.86 | 0.62 | 2.10 | 0.83 |  |
| 175_L | Mouse | Large Intestine | Burn | 3D | 28 | 2.67 | 0.67 | 2.23 | 0.87 |  |
| 176_L | Mouse | Large Intestine | Burn | 3D | 30 | 2.86 | 0.62 | 2.12 | 0.86 |  |
| 150_L | Mouse | Large Intestine | Sham |  | 30 | 2.86 | 0.60 | 2.03 | 0.82 |  |
| 151_L | Mouse | Large Intestine | Sham |  | 31 | 2.96 | 0.63 | 2.16 | 0.84 |  |
| 152_L | Mouse | Large Intestine | Sham |  | 32 | 3.06 | 0.63 | 2.17 | 0.85 |  |
| 167_L | Mouse | Large Intestine | Sham |  | 28 | 2.67 | 0.64 | 2.13 | 0.84 |  |
| 168_L | Mouse | Large Intestine | Sham |  | 28 | 2.67 | 0.64 | 2.13 | 0.83 |  |
| 154_S | Mouse | Small Intestine | Burn | 1D | 26 | 2.47 | 0.57 | 1.86 | 0.79 |  |
| 155_S | Mouse | Small Intestine | Burn | 1D | 26 | 2.47 | 0.56 | 1.83 | 0.79 |  |
| 157_S | Mouse | Small Intestine | Burn | 1D | 30 | 2.86 | 0.68 | 2.32 | 0.87 |  |
| 158_S | Mouse | Small Intestine | Burn | 1D | 34 | 3.26 | 0.44 | 1.56 | 0.61 |  |
| 159_S | Mouse | Small Intestine | Burn | 1D | 26 | 2.47 | 0.45 | 1.47 | 0.70 |  |
| 171_S | Mouse | Small Intestine | Burn | 3D | 32 | 3.06 | 0.22 | 0.76 | 0.40 |  |
| 172_S | Mouse | Small Intestine | Burn | 3D | 36 | 3.46 | 0.49 | 1.76 | 0.72 |  |
| 173_S | Mouse | Small Intestine | Burn | 3D | 27 | 2.57 | 0.52 | 1.73 | 0.75 |  |
| 175_S | Mouse | Small Intestine | Burn | 3D | 34 | 3.26 | 0.51 | 1.79 | 0.75 |  |
| 176_S | Mouse | Small Intestine | Burn | 3D | 33 | 3.16 | 0.38 | 1.34 | 0.66 |  |
| 150_S | Mouse | Small Intestine | Sham |  | 38 | 3.65 | 0.62 | 2.27 | 0.85 |  |
| 151_S | Mouse | Small Intestine | Sham |  | 30 | 2.86 | 0.37 | 1.25 | 0.53 |  |
| 152_S | Mouse | Small Intestine | Sham |  | 29 | 2.77 | 0.42 | 1.42 | 0.58 |  |
| 167_S | Mouse | Small Intestine | Sham |  | 28 | 2.67 | 0.48 | 1.60 | 0.67 |  |
| 168_S | Mouse | Small Intestine | Sham |  | 29 | 2.77 | 0.50 | 1.68 | 0.71 |  |
|  |  |  |  |  |  |  |  |  |  |  |
| * All analyses were based on rarefied datasets of 25,000 sequences. Diversity indices were calculated within the software package Primer6. S = Total number of families; N = Total number of sequences analyzed; d = Margalef Richness; J' = Pielou's evenness; H' = Shannon index (log e); 1-Lambda' = Simpson's index. | | | | | | | | | | |
