# Supplementary material for: Burn Injury Alters the Intestinal Microbiome and Increases Gut Permeability and Bacterial Translocation
Source: PLoS One. 2015 Jul 8;10(7):e0129996. doi: 10.1371/journal.pone.0129996 (PMC4496078; doi:10.1371/journal.pone.0129996)
Supplement: S2 Table — (DOCX) [file pone.0129996.s002.docx]

| **Supplemental Table 2: Group differences in Alpha diversity indices*** | | | | | |
| --- | --- | --- | --- | --- | --- |
|  |  |  |  |  |  |
|  | S | d | J' | H'(loge) | 1-Lambda' |
| **Control-vs-Burn** |  |  |  |  |  |
| Av-Control | 32.63 | 3.12 | 0.53 | 1.85 | 0.76 |
| Av-Burn | 27.60 | 2.63 | 0.50 | 1.67 | 0.75 |
| TTEST | 0.01 | 0.01 | 0.46 | 0.20 | 0.77 |
|  |  |  |  |  |  |
| **SI_Sham-vs-Burn1d** |  |  |  |  |  |
| Av-SI-Sham | 30.80 | 2.94 | 0.48 | 1.64 | 0.67 |
| Av-SI-Burn-1d | 30.40 | 2.90 | 0.64 | 2.18 | 0.84 |
| TTEST | 0.85 | 0.85 | 0.02 | 0.03 | 0.03 |
|  |  |  |  |  |  |
| **SI_Sham-vs-Burn3d** |  |  |  |  |  |
| Av-SI-Sham | 30.80 | 2.94 | 0.48 | 1.64 | 0.67 |
| Av-SI-Burn-3d | 32.40 | 3.10 | 0.43 | 1.48 | 0.66 |
| TTEST | 0.52 | 0.52 | 0.48 | 0.54 | 0.91 |
|  |  |  |  |  |  |
| **SI_Burn1d-vs-Burn3d** |  |  |  |  |  |
| Av-SI-Burn-1d | 28.40 | 2.71 | 0.54 | 1.81 | 0.75 |
| Av-SI-Burn-3d | 32.40 | 3.10 | 0.43 | 1.48 | 0.66 |
| TTEST | 0.11 | 0.11 | 0.15 | 0.22 | 0.27 |
|  |  |  |  |  |  |
| **LI_Sham-vs-Burn1d** |  |  |  |  |  |
| Av-LI-Sham | 29.80 | 2.84 | 0.63 | 2.13 | 0.84 |
| Av-LI-Burn-1d | 31.60 | 3.02 | 0.67 | 2.32 | 0.88 |
| TTEST | 0.17 | 0.17 | 0.00 | 0.00 | 0.00 |
|  |  |  |  |  |  |
| **LI_Sham-vs-Burn3d** |  |  |  |  |  |
| Av-LI-Sham | 29.80 | 2.84 | 0.63 | 2.13 | 0.84 |
| Av-LI-Burn-3d | 29.40 | 2.80 | 0.62 | 2.10 | 0.83 |
| TTEST | 0.67 | 0.67 | 0.70 | 0.55 | 0.77 |
|  |  |  |  |  |  |
| **LI_Burn1d-vs-Burn3d** |  |  |  |  |  |
| Av-LI-Burn-1d | 31.60 | 3.02 | 0.67 | 2.32 | 0.88 |
| Av-LI-Burn-3d | 29.40 | 2.80 | 0.62 | 2.10 | 0.83 |
| TTEST | 0.06 | 0.07 | 0.01 | 0.00 | 0.03 |
|  |  |  |  |  |  |
| * All analyses were based on rarefied datasets of 25,000 sequences. Diversity indices were calculated within the software package Primer6. S = Total number of families; N = Total number of sequences analyzed; d = Margalef Richness; J' = Pielou's evenness; H' = Shannon index (log e); 1-Lambda' = Simpson's index. TTEST represents two-tailed TTEST with unequal variance. TTESTs with p<0.05 are highlighted in green. | | | | | |
